# Enhancing Open-Space Gas Detection Limit: A Novel Environmentally Adaptive Infrared Temperature Prediction Method for Uncooled Spectroscopy

**DOI:** 10.3390/s24227173

**Published:** 2024-11-08

**Authors:** Guoliang Tang, Fang Ding, Dunping Li, Bangjian Zhao, Chunlai Li, Jianyu Wang

**Affiliations:** 1 Hangzhou Institute for Advanced Study, University of Chinese Academy of Sciences, Hangzhou 310024, China; tangguoliang@mail.sitp.ac.cn (G.T.); dingfang22@mails.ucas.ac.cn (F.D.); lidunping23@mails.ucas.ac.cn (D.L.);; 2 University of Chinese Academy of Sciences, Beijing 100049, China; 3 Key Laboratory of Space Active Opto-Electronics Technology, Shanghai Institute of Technical Physics, Chinese Academy of Sciences, Shanghai 200083, China

**Keywords:** uncooled infrared spectroscopy, gas cloud imaging, temperature compensation, detection limit enhancement

## Abstract

Gas cloud imaging with uncooled infrared spectroscopy is influenced by ambient temperature, complicating the quantitative detection of gas concentrations in open environments. To solve the aforementioned challenges, the paper analyzes the main factors influencing detection errors in uncooled infrared spectroscopy gas cloud imaging and proposes a temperature correction method to address them. Firstly, to mitigate the environmental effects on the radiative temperature output of uncooled infrared detectors, a snapshot-based, multi-band infrared temperature compensation algorithm incorporating environmental awareness was developed. This algorithm enables precise infrared radiation prediction across a wide operating temperature range. Validation tests conducted over the full temperature range of 0 °C to 80 °C demonstrated that the prediction error was maintained within ±0.96 °C. Subsequently, temperature compensation techniques were integrated, resulting in the development of a comprehensive uncooled infrared spectroscopy gas cloud imaging detection method. Ultimately, the detection limits for SF6, ethylene, cyclohexane, and ammonia were enhanced by 50%, 33%, 25%, and 67%, respectively.

## 1. Introduction

Gas leaks can lead to serious safety incidents, making the rapid and accurate detection of leaks essential for preventing accidents. Infrared spectroscopy imaging technology enables remote, non-contact gas detection, making it a powerful tool for monitoring gas leaks and issuing safety warnings in complex environments [1]. However, traditional cooled infrared detectors are limited in portable and dynamic monitoring applications due to their large size, high cost, system complexity, and the challenges associated with operation, maintenance, and prolonged cooling requirements. In contrast, uncooled infrared detectors have effectively addressed these challenges, leading to significant advancements in portable devices and real-time, all-weather dynamic monitoring [2].

Despite these advancements, uncooled infrared detectors still face limitations in accuracy and sensitivity when performing quantitative gas concentration detection in open environments. Researchers have enhanced spectral selectivity and temperature compensation methods, enabling the identification and quantification of multiple gases, including SF6, methane, and ammonia. These enhancements have significantly improved the practicality and reliability of uncooled infrared gas detection in both environmental monitoring and industrial applications [3,4,5]. However, to achieve simultaneous gas detection and species identification, it is necessary to capture both spatial and spectral information in a single snapshot, which requires snapshot spectral imaging technology [6,7]. In response to this need, our team developed and tested an infrared spectroscopy gas cloud camera between 2022 and 2023, successfully achieving gas concentration retrieval [8,9]. However, the instrument is still susceptible to interference from ambient temperature, which affects its sensitivity and accuracy.

The non-uniformity of the detector array and environmental variations, particularly changes in ambient temperature, are the primary causes of interference in temperature signal detection. In the past, cameras typically used integrated thermoelectric coolers (TECs) to maintain a constant temperature for the focal plane array (FPA), and an electronically controlled shutter was employed as a reference to prevent sensor parameters such as offset voltage and responsivity from drifting due to environmental temperature changes. However, this approach comes with notable drawbacks. First, the use of a TEC results in high power consumption, and more critically, the temperature control by a TEC causes frequent oscillations around the target temperature for the FPA, which affects the stability of detection [10]. Furthermore, shutter corrections are only performed when the device detects temperature changes beyond a certain threshold, meaning that temperature disturbances within this threshold are overlooked, leading to signal interference.

To address this issue, Budzier and Gerlach (2015) proposed a new shutterless compensation method based on a calibration procedure [11,12]. This method compensates for the effects of environmental temperature changes by detecting real-time temperature fluctuations of the device and determining corresponding correction parameters. However, most existing methods focus solely on the temperature changes of the FPA, while the device’s response is also influenced by other factors, such as the optical components and structural elements.

Therefore, we propose a temperature correction method for an uncooled infrared hyperspectral gas cloud camera that is applicable in various environments. This method integrates a model that accounts for multiple factors, including the camera casing, FPA, flat-field support, and environmental temperature variations. By optimizing the temperature correction points, this method effectively minimizes the signal disturbances caused by ambient temperature fluctuations and internal heating of the device.

As a result, the system can consistently and accurately reconstruct the radiative properties of the imaged scene, even under dynamic environmental conditions. Testing across the full temperature range of 0 °C to 80 °C showed that the error remained within ±0.96 °C. Furthermore, by integrating temperature compensation techniques, we developed a quantitative detection method for uncooled infrared spectral gas cloud imaging. The detection limits for SF6, ethylene, cyclohexane, and ammonia were improved by 50%, 33%, 25%, and 67%, respectively.

## 2. Infrared Radiation Measurement and Gas Detection

### 2.1. Detection Energy Model of Thermal Infrared Spectral Imaging

The core principle of infrared gas detection technology is that it describes the relationship between a substance’s absorption of light and its concentration. In the infrared spectrum range, specific gas molecules absorb infrared radiation energy corresponding to their molecular vibration and rotational energy levels [13]. By measuring these changes in energy, the concentration of the gas can be quantitatively determined. In infrared optoelectronic physics theory, the spectral radiance of a blackbody is typically denoted by M(λ,  T):

(1) $$M\left(\lambda ,T\right)=\frac{2\pi hc^{2}}{\lambda^{5}}\frac{1}{e^{\frac{ch}{\lambda kT}}-1}$$

$M_{\lambda }$ represents the spectral radiance of a blackbody, with units of $W\cdot cm^{-2}\cdot \mu m^{-1}$; *T* denotes the absolute temperature of the blackbody, typically referenced at 300 K for performance calculations; *λ* is the wavelength; *h* is Planck’s constant with a value of $6.626196\times 10^{-34}$ $W\cdot s^{2}$; c is the speed of light in a vacuum, valued at $2.997925\times 10^{10}\;cm\cdot s^{-1}$; and *k* is the Boltzmann constant, with a value of $1.380649\times 10^{-23}\;J\cdot K^{-1}$. The equation can be transformed as follows:

(2) $$M\left(\lambda ,T\right)=\frac{c_{1}}{\lambda^{5}}\frac{1}{e^{\frac{c_{2}}{\lambda T}}-1}$$

$c_{1}$ is the first radiation constant, with a value of $3.7418\times 10^{-12}\;Wm^{2}$, and $c_{2}$ is the second radiation constant, with a value of 1.4388 cmk.

Generally, the blackbody can be considered as a Lambertian radiator. The expression for the spectral radiance *L*(*λ*,*T*) of a blackbody is given by Planck’s law:

(3) $$L\left(\lambda ,T\right)=\frac{M\left(\lambda ,T\right)}{\pi }=\frac{c_{1}}{\pi \times \lambda^{5}}\frac{1}{e^{\frac{c_{2}}{\lambda T}}-1}$$

L(λ,T) represents the spectral radiance at wavelength *λ* and temperature *T*, *T* is the absolute temperature of the blackbody.

**Figure 1:**
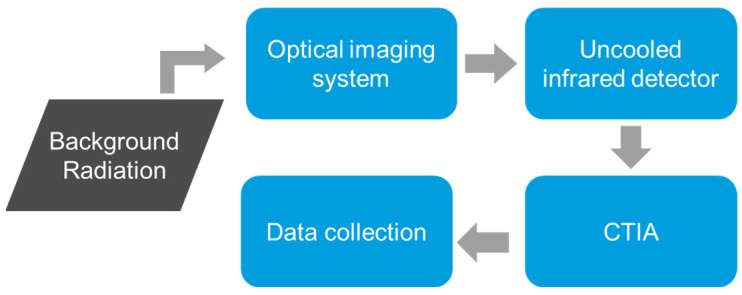
Full information flow diagram of conventional infrared imaging system model.

For imaging systems, the transmission path is typically modeled as a homogeneous atmosphere, with the transmission efficiency denoted by τ(λ). The information flow in the detection model of the imaging system is illustrated in Figure 1, where the infrared signal, after passing through the photosensitive material, is converted into a digital signal by a Capacitive Trans-impedance Amplifier (CTIA) [14]. This amplifier effectively translates the optical signal into an electrical form for further processing. The energy received by the sensor consists of the following components: (1) thermal radiation emitted by the observed background scene, (2) thermal radiation from surrounding objects reflected by the observed background, (3) solar radiation reflected by the observed background, and (4) atmospheric path radiance $L_{P}(\lambda ,\;T)$.

In the thermal infrared spectral range, (3) the solar radiation reflected by the observed background can be neglected, and (1) and (2) can be combined into the radiance emitted by the observed background, denoted as $L_{g}(\lambda ,\;T)$. Consequently, the radiance at the sensor’s entrance pupil can be expressed as follows:

(4) $$L\;\left(\lambda ,T\right)=L_{g\;}\left(\lambda ,T\right)\times \tau \;\left(\lambda \right)+L_{p}\;\left(\lambda ,T\right)\;=\left\{\varepsilon \;\left(\lambda \right)\times L_{back}\;\left(\lambda ,T\right)+\left(1-\varepsilon \;\left(\lambda \right)\right)\times L_{a}\;\left(\lambda ,\;T\right)\right\}\times \tau \;\left(\lambda \right)+L_{p}\left(\lambda ,T\right)$$

If the observed background is a blackbody, only the thermal radiation emitted by the background itself is present, which represents the ideal target infrared image captured by a conventional infrared imaging system. Under the assumption that the observed background is a blackbody, the equation simplifies to the following:

(5) $$L\;\left(\lambda ,T\right)=L_{back}\;\left(\lambda ,T\right)\times \tau \;\left(\lambda \right)+L_{p}\left(\lambda ,T\right)$$

The calculation within the parentheses is consistent with the previously derived spectral radiance $L_{g}$(λ) and depends only on the temperature *T*, following the distribution by wavelength, expressed as follows:

The $L_{g}$(λ) depends solely on the temperature *T*:

(6) $$L_{g}\left(\lambda ,T\right)=\frac{M\left(\lambda ,T\right)}{\pi }=\frac{c_{1}}{\pi \lambda^{5}}\frac{1}{e^{\frac{c_{2}}{\lambda T}}-1}$$

Under close-range conditions or favorable atmospheric conditions, the atmospheric path radiance can be approximated as negligible. Given the spectral radiance $L_{g}$(λ), the sensor’s optical aperture area *A* (cm^2^), the instantaneous field of view solid angle *Ω* (Sr), and the transmission efficiency *τ*(*λ*), the radiative power $P_{tar}(\lambda ,T)$ received by the sensor’s pixel can be calculated as follows:

(7) $$P_{tar}\;\left(\lambda ,T\right)=L_{g}\;\left(\lambda ,T\right)\times A\times \Omega^{2}\times \tau \;\left(\lambda \right)\;=\frac{M\;\left(\lambda ,\;T\right)}{\pi }\times \frac{\pi {D_{o}}^{2}}{4}\times \Omega^{2}\times \tau \left(\lambda \right)=\frac{1}{4}M\;\left(\lambda ,T\right)\times {D_{o}}^{2}\times \Omega^{2}\times \tau \;\left(\lambda \right)$$

$D_{o}$ represents the effective aperture diameter of the optical system, *Ω* is the solid angle of the sensor’s instantaneous field of view, and τ(λ) denotes the transmission efficiency.

The characteristic spectral absorption of the gas affects the transmission efficiency *τ* (*λ*) in gas detection, with varying absorption properties across different wavelengths. This variation enables the identification of different gas species. By analyzing the depth and intensity of the absorption, it is possible to perform quantitative concentration measurements. This approach represents the fundamental physical mechanism behind gas detection using active and passive optical methods, based on the concept of optical fingerprints [15].

The equation can be further simplified by expressing it in terms of the optical relative aperture $F^{\#}$ and the detector pixel size *d*, yielding the following:

(8) $$P_{tar}(\lambda ,T)=\frac{1}{4}M(\lambda ,T)\times \tau (\lambda )\times (\frac{d}{F^{\#}}{)}^{2}$$

The target radiative power ${\;P}_{tar}\;(\lambda ,T)$ is determined by the transmission efficiency *τ*(*λ*) and the spectral radiance of the observed background $L_{g\;}(\lambda ,T)$. By multiplying these factors by the corresponding detection spectral bandwidth, the power received by the detector pixel can be determined. The equation also indicates that, for the sensor, the received power depends on the optical efficiency τ (λ), the spectral sampling interval Δλ, and the optical relative aperture $F^{\#}$. For the detector, the received power is influenced by the pixel size *d*.

### 2.2. The Principle of Infrared Gas Detection

**Figure 2:**
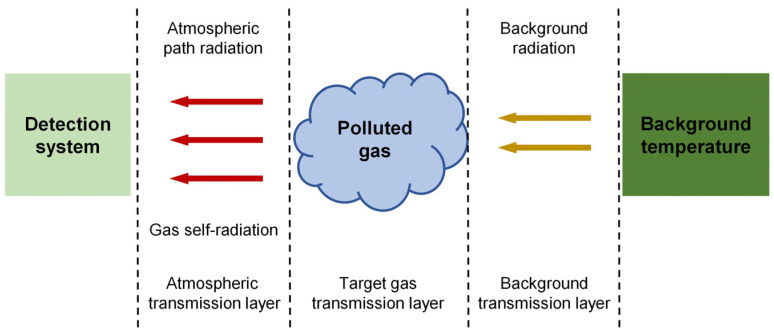
Three-layer atmospheric radiation transfer detection model.

When a pollutant gas is introduced into the transmission path, the information flow in the detection model is depicted in Figure 2. The background radiation temperature corresponds to the target imaging area in a conventional imaging system. Let $L_{off}\;(\lambda_{i},T)$ represent the radiance from the background that reaches the sensor’s entrance pupil directly through the atmosphere, without passing through the pollutant gas [16].

Let $L_{on}\;(\lambda_{i},T)$ represent the radiance from the background that reaches the sensor’s entrance pupil after passing through the pollutant gas, which equals $L_{off}(\lambda_{i},T)$ in the absence of the pollutant gas. The radiative transfer equation for the gas path, based on the direction of radiation transmission, is expressed as follows:

(9) $$\left\{\begin{array}{c} L_{on}\;\left(\lambda_{i},T\right)=L_{bg}\;\left(\lambda_{i},T\right){\;t}_{i}\;\left(\lambda_{i}\right)\;t_{2\;}\left(\lambda_{i}\right){\;t}_{g\sigma \;}\left(\lambda_{i}\right)+L_{g\sigma }\;\left(\lambda_{i},T\right)\;t_{2}\;\left(\lambda_{i}\right)+L_{p}\;\left(\lambda_{i},T\right)\\ L_{of}\;\left(\lambda_{i},T\right)=L_{bg\;}\left(\lambda_{i},T\right)\;t_{1\;}\left(\lambda_{i}\right)\;t_{2\;}\left(\lambda_{i}\right)+L_{p}\left(\lambda_{i},T\right)\\ L_{\mathrm{ga}}\;\left(\lambda_{i},T\right)=B\;\left(\lambda_{i},T_{g\sigma }\right)\left[1-\tau_{g\pi }\left(\lambda_{i}\right)\right] \end{array}\right.$$

$L_{gas}\;(\lambda ,\;T)\;$ represents the spectral radiance of the pollutant gas itself, which is calculated based on the blackbody radiation at the gas temperature $T_{gas}$ and the gas’s emissivity $B\;(\lambda_{i},T_{gas})$. $L_{bj}\;(\lambda_{i},T)$ represents the actual background radiance. $L_{p}\;(\lambda_{i},T)$ denotes the radiance along the gas path. $\tau_{gas}\;(\lambda_{i})$ represents the transmittance of the pollutant gas, which can be calculated using the Beer–Lambert law. $t_{1}\;(\lambda_{i})$ and $t_{2}\;(\lambda_{i})$ represent the atmospheric attenuation transmittance in the background transmission layer and the atmospheric transmission layer, respectively.

In practical scenarios, the distance between the target gas cloud and background objects, such as buildings, is typically small. The distance between the background transmission layer and the target gas transmission layer is relatively short compared to the atmospheric transmission layer. Consequently, the atmospheric effects within the background and target gas transmission layers can be considered negligible, allowing Equation (9) to be simplified as follows:

(10) $$\left\{\begin{array}{l} L_{on}\;\left(\lambda_{i},T\right)=L_{bg\;}\left(\lambda_{i},T\right)\tau_{gas}\;(\lambda_{i})+L_{gas}\;(\lambda_{i},T)\\ L_{off}\;(\lambda_{i},T)=L_{bg}\;(\lambda_{i},T)\\ L_{gas\;}(\lambda_{i},T)=B\;(\lambda_{i},T_{gas})\;[1-\tau_{gas}(\lambda_{i})] \end{array}\right.$$

the radiance values $L_{on}$ and $L_{off}\;$ will be attenuated to $L_{on2}$ and $L_{off2}$ due to the limitations imposed by the lens, filter transmittance, and detector response. These attenuated radiance values are then converted into data values reflecting the intensity of the radiation. Consequently, the difference between the background radiance without passing through the pollutant gas and the background radiance after passing through the pollutant gas is given by the following:

(11) $$\left\{\begin{array}{c} \Delta L=L_{off}\cdot \tau_{z}-L_{on}\cdot \tau_{z}=\tau_{z}\left(1-\tau_{gas}\right)\left(L_{bg}-B\right)\\ \tau_{z}=\tau_{a}\cdot \tau_{c}\cdot \tau_{svf} \end{array}\right.$$

$\tau_{z}$, $\tau_{a}$, $\tau_{c}$, and $\tau_{srf}$ represent the total system response, filter transmittance, optical lens transmittance, and detector response, respectively.

## 3. Temperature Compensation Model

The response characteristics of uncooled infrared detectors are susceptible to fluctuations in ambient temperature, significantly affecting the accuracy and stability of subsequent temperature measurements [17]. Developing radiation calibration data acquisition and processing methods that adapt to different operating temperatures and establishing an accurate mapping between the grayscale output of uncooled infrared detectors and the radiative temperature is crucial for achieving high-precision infrared temperature measurements in complex environments. Here, we analyze the impact of ambient temperature and shutter temperature on detector response through laboratory calibration methods, propose corresponding correction models, and summarize a reliable absolute radiation calibration method for uncooled infrared detectors.

### 3.1. Impact of Ambient Temperature on Detector Response

This part evaluates the impact of ambient temperature on detector response by testing response variations under varying temperature conditions, conducting a correlation analysis, and determining correction coefficients.

#### 3.1.1. Thermal Chamber Testing Experiment

**Figure 3:**
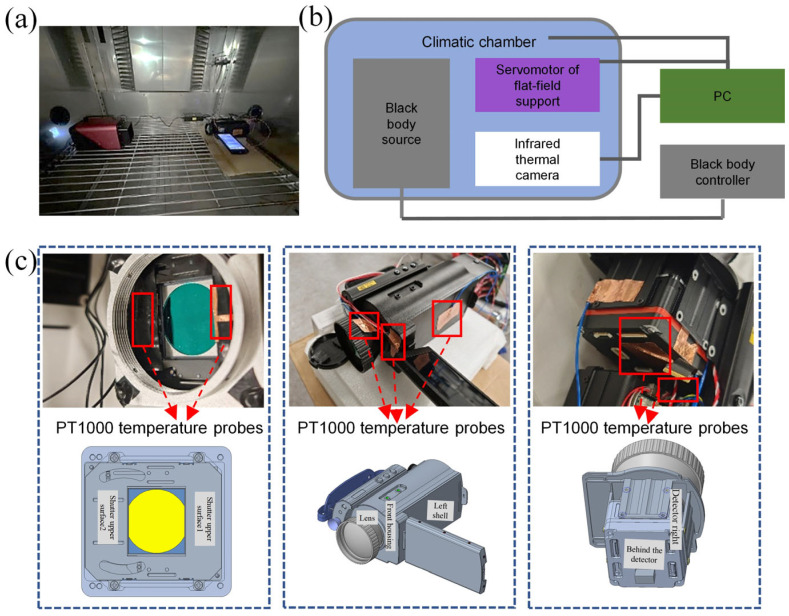
Thermal chamber testing experiment (**a**) experimental scheme, (**b**) actual picture, and (**c**) temperature measurement point location.

The equipment was placed in a thermal chamber (Figure 3a), which can provide a continuous varying temperature environment, thereby altering the equipment’s operating temperature (Figure 3b). The electrical losses generated by the digital signal processor FPGA and sensor array during operation lead to an uneven temperature distribution within the uncooled imaging device. The loss creates a significant thermal gradient between the sensor and the inner side of the camera. To accurately monitor temperature changes, a commercial thermometer (model MIK-R8000A, from Hangzhou Meacon Automation Technology Co., Ltd., Hangzhou, China) was used for temperature measurements. Inside the camera, eight PT1000 temperature probes were installed at key locations, including the camera core, focal plane, shutter, detector housing, and lens (Figure 3c). These probes have different time responses to ambient temperature changes and work in conjunction with internal temperature measurement points to monitor the internal FPA temperature and ambient temperature of the infrared detector IRay FTII1280 in real-time, ensuring comprehensive and accurate temperature monitoring. The equipment information is provided in Table 1. In Table 1, the detector type utilizes vanadium oxide (VOx), a material commonly used in uncooled infrared focal plane arrays due to its high temperature coefficient of resistance (TCR). VOx detectors are highly sensitive to infrared radiation, allowing them to operate effectively at ambient temperatures without the need for cryogenic cooling. This makes them a cost-effective and reliable choice for thermal imaging applications.

A blackbody (Model: Medium and High Temperature Blackbody from Optoelectronics Technology Co., Ltd., Jiyuan, Hangzhou, China) was selected as the reference heat source in the experiment. The temperature was set to 50 °C to minimize the response of the blackbody to changes in the external environment and to ensure the stability of the experimental data. The equipment was placed in a thermal chamber to collect infrared images of the blackbody heat source under dynamically changing external working temperature conditions. The temperature setting of the thermal chamber was gradually increased from 10 °C to 50 °C, and then decreased from 50 °C to 10 °C, forming a complete temperature cycle. The duration of each cycle was set to one hour, and the experiment was carried out twice in a row for a total of 4 h to fully evaluate the response characteristics of the equipment under different ambient temperatures.

#### 3.1.2. Correlation Calculation

**Figure 4:**
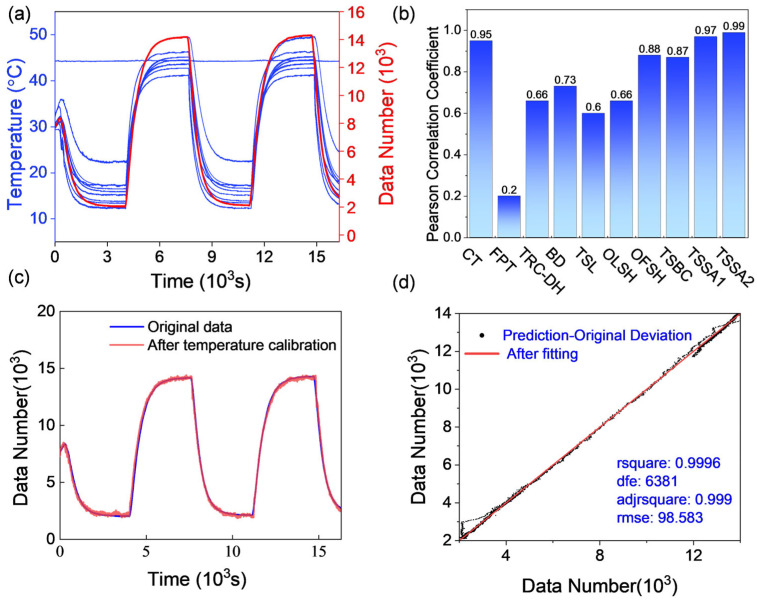
(**a**) Output value changes of detector (red curve) and 10 temperature data at the corresponding time; (**b**) correlation between temperature change and detector response, from left to right: core temperature (CT), focal plane temperature (FPT), top-right corner of detector housing (TRC-DH), back of detector (BD), top side of lens (TSL), outer left side of housing (OLSH), outer front Side of housing (OFSH), top surface of battery compartment (TSBC), top surface of shutter assembly 1 (TSSA1), and top surface of shutter assembly 2 (TSSA2); (**c**) original temperature and fitting curves; and (**d**) original value and correlation fitting curve.

In the temperature experiment, the changes of 10 temperature points were measured to explore the optimal temperature measurement point, providing a reliable basis for subsequent research and development. The results show that the output value change of the detector (red curve) and the 10 temperature measurement data at the corresponding time have a relatively obvious consistency (Figure 4a). The Pearson correlation coefficient was used to characterize the correlation between temperature changes and detector response [18]. The results indicate a positive correlation between the detector’s response data and temperature, meaning that as the temperature increases, the DN value (similar to the camera signal count) also increases (Figure 4b). Notably, the correlation between the top surface temperature and the DN value exceeded 95% (97% and 99%, respectively), indicating that the shutter component’s temperature significantly affects the DN value. Additionally, the core temperature directly reflects the overall operating temperature of the detector, and the experimental results confirm that the core’s output value is directly related to its operating temperature. It is important to note that although the measured FPA (focal plane array) temperature correlation was only 20%, this does not indicate that the FPA temperature is unrelated to the DN value. On the contrary, this result may be due to the FPA’s temperature control function being enabled during the test, partially eliminating the temperature effect, which is why the correlation is lower.

#### 3.1.3. Model Confirmation and Parameter Calculation

A linear fitting analysis was performed on the relationship between the ambient temperature variable of the experimental data and the detector output. The temperature data are the independent variable, and the detector output value is the dependent variable, forming a multivariate dataset for fitting. The correlation results between each column of independent variables and the dependent variable show that there is a strong positive correlation between each column of independent variables and the dependent variable, and the correlation coefficient R is close to 1, including the movement temperature (R = 0.95), the upper surface of the shutter assembly (R = 0.97), and the second upper surface of the shutter assembly (R = 0.99). The above three temperature points are selected for linear regression fitting, and the fitting equation can be expressed as follows:

(12) $$y=\beta_{0}+\beta_{1}X_{1}+\beta_{2}X_{2}+\beta_{3}X_{3}$$

where *y* is the dependent variable, $\beta_{0}$ is the intercept, and $\beta_{1}{,\beta }_{2}$, and $\beta_{3}$ are the regression coefficients of the independent variables $X_{1},\;\;X_{2}$, and $X_{3}$ respectively. The specific regression coefficients and intercept were obtained through fitting, indicating that the contributions of each variable to the dependent variable are different. The fitting results show that the model effectively reflects the linear relationship between the independent and dependent variables. After determining the parameters, the fitting formula is as follows:

(13) $$y=-5486.7137+(564.8505\times X_{1})+(-486.2607\times X_{2})+\left(439.7826\times X_{3}\right)$$

The fitted parameters are introduced into the temperature curve. The fitting curve is highly consistent with the original data (Figure 4c). Figure 4d presents the analysis of the fitting curve, which shows that the predicted value of the model is consistent with the actual value, and the calculated residual is 98.583 (RMES1).

### 3.2. Shutter Compensation Method

**Figure 5:**
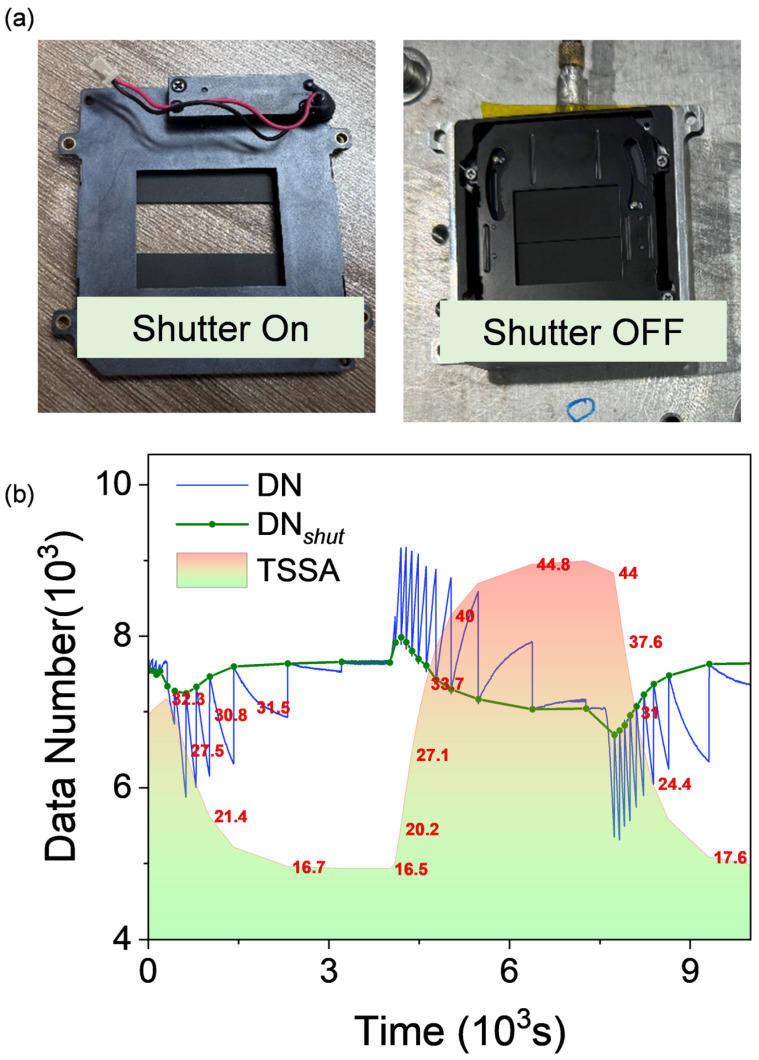
(**a**) Shutter operation (opening and closing), (**b**) ambient temperature, detector response, and the detector’s response value at the time of shutter correction.

The shutter mainly ensures that the response voltage of the detector remains within the normal range, preventing the response of some pixels from exceeding the range, causing the analog-to-digital converter to malfunction. In addition, a uniform radiation surface (similar to a uniform blackbody) can be provided for calibrating the detector (Figure 5a). However, during quantitative detection, the presence of the shutter may affect the consistency of the detector response, making it difficult for the user to accurately calculate the target temperature based on a unified temperature reference. Therefore, establishing a relationship model between the shutter and the blackbody response is important to ensure the accuracy and consistency of the measurement results.

#### 3.2.1. The Influence of Shutter Temperature on Detector Response

Objects above absolute zero have thermal radiation, and so does the shutter. During imaging, the shutter blocks the lens from focusing the light signal entering the system, so that the detector’s response only corresponds to the shutter’s thermal radiation to complete the shutter correction. The DN value received by the detector can be expressed as follows:

(14) $$DN_{shut}=f_{1}\;\left(Tmp_{shut}\right)$$

where $f_{1}()$ represents a simplified radiation detection function at the shutter, indicating the relationship between the target temperature radiation and DN value. The corrected output by the uncooled camera is the detector’s response value to the target based on the shutter temperature, so the obtained data reflect the DN value at the moment of correction:

(15) $$DN_{shut_{obj}}=f_{1}\;\left(Tmp_{obj}-Tmp_{shut}+Tmp_{lens}\right)+DN_{shut}$$

where ${DN}_{shut\_obj}$ is the corrected value to the target, $Tmp_{obj}$ is the target temperature, $Tmp_{shut}$ is the shutter temperature, $Tmp_{lens}$ is the lens temperature, and α,  β,  γ are correction coefficients for different temperatures, with α  obtained through radiation calibration, as described in Section 3.2.2. $DN_{shut}$ is the detector’s signal output when the target temperature equals the shutter temperature (avoiding negative DN values). Ignoring the non-linear response during detection, the radiation calibration can be expressed as follows:

(16) $$DN_{shut_{obj}}=\alpha \cdot Tmp_{obj}-\beta \cdot Tmp_{shut}+\gamma \cdot Tmp_{lens}+B$$

To validate the correlation between shutter temperature variations and detector response values, the relationship between shutter temperature and detector response under varying temperature conditions was investigated. The experiment was conducted in a thermal chamber, utilizing a constant-temperature blackbody and an uncooled imaging device. The test process is basically the same as the previous steps. In order to better simulate the actual situation of the device, the shutter correction function was turned on during the experiment and calibrated every five minutes. A mechanical shutter is used to block the field of view and automatically perform timed image non-uniformity correction to ensure that the imaging device maintains optimal performance during continuous operation.

For a stable-temperature blackbody target, the detector’s response value changes continuously over time, as shown in Figure 5b. The blue curve represents the variation in the response value when the blackbody temperature remains constant, while the color-filled area indicates the temperature fluctuations in the thermal chamber during the experiment. The green curve in the figure shows the device’s response to the blackbody after shutter correction, obtained through data processing. The experiment also recorded temperature data from 10 points, revealing that fluctuations in the response value during shutter correction are also driven by temperature changes, with the response value being inversely correlated with ambient temperature. By extracting the temperature data and DN value responses at the shutter correction moments and fitting them, the relationship between the detector response and shutter temperature was derived, as detailed in Section 3.2.2.

#### 3.2.2. Radiometric Calibration

**Figure 6:**
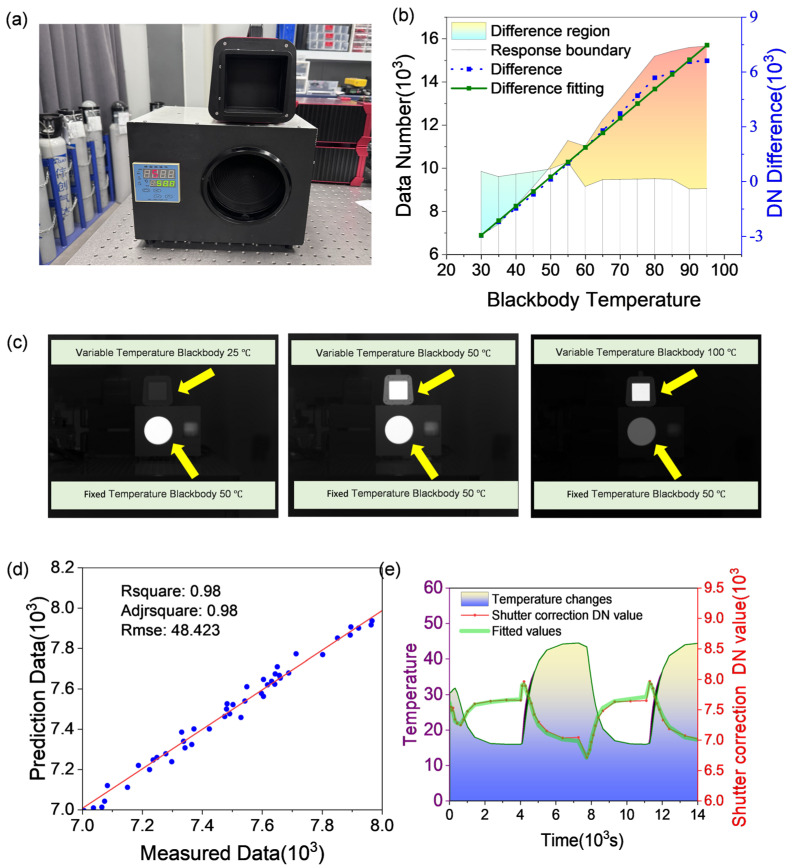
(**a**) Photograph of the dual blackbody setup, (**b**) detector response variation for the dual blackbodies, (**c**) sample image collected during the experiment, (**d**) comparison between fitted linear coefficients and actual values, and (**e**) performance of the shutter temperature compensation model.

The radiometric calibration aims to calibrate and adjust the infrared thermal imaging system to ensure that its output data accurately reflect the radiative intensity. By using a radiation source with a known spectral intensity, the infrared system is calibrated to establish a relationship between the grayscale values in infrared images and the radiative temperature. To address the temperature drift in uncooled detectors, a dual blackbody calibration method was employed (Figure 6a). The process is as follows: two standard blackbody radiation sources, one with a fixed temperature and the other with a variable temperature, were placed directly in front of the system. The fixed-temperature blackbody was set to 50 °C to provide a reference response value. By comparing the measured values with this reference response, temperature drift data for the uncooled detector were obtained, which were then used for temperature drift compensation in subsequent processing. The variable-temperature blackbody was gradually heated from 25 °C to 100 °C, with 5 °C increments serving as temperature points. At each temperature point, 150 frames of images were captured to ensure that the system’s response at different radiation intensities was thoroughly recorded. Some of the experimental data collected are shown in Figure 6c, where the upper blackbody represents the variable-temperature blackbody, and the lower one represents the fixed-temperature blackbody.

The *DN* represents the digital value in the infrared image, while a and b are coefficients obtained through experimental fitting. The fitting and calculation of the corresponding radiative intensity are then performed using the DN values from the fixed-temperature and variable-temperature blackbodies. The signal is derived from the difference between the fixed-temperature and variable-temperature blackbodies to prevent the introduction of the temperature drift noise. The deviation between the fixed and variable blackbodies is shown in Figure 6b. The radiometric measurement formula is obtained through linear fitting, as shown in Equation (17). The comparison between the fitted linear coefficients and actual values, with a root mean square error (RMSE2) of 48.423, is presented in Figure 6d.

(17) y=160.1∗DN+b

The radiation calibration is the slope of the curve α=160.1, and the detector’s DN_base=8000 (device manual). The complete correction parameters are α = 160.1, β = 192.43, and γ = 154.25. The correction model formula is shown in Equation (18). Figure 6e shows the comparison between the actual shutter response data and the model calculation data after the shutter correction model parameters are improved.

(18) $$DN_{shut_{obj}}=160.1\cdot Tmp_{obj}-192.43\cdot Tmp_{shut}+154.25\cdot Tmp_{lens}+8000$$

### 3.3. The Result of Temperature Correction

**Figure 7:**
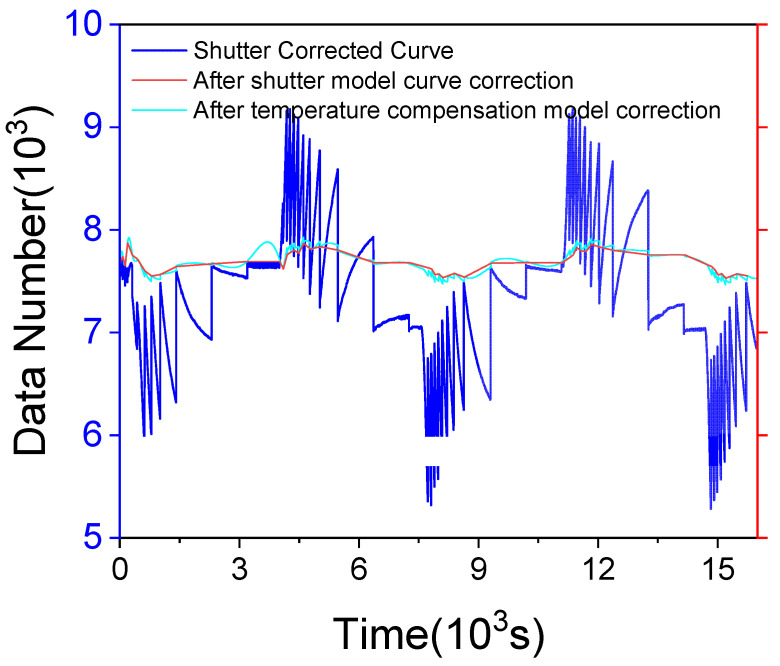
Corrected temperature response.

By implementing temperature and shutter corrections, the system realized significant improvements in calibration accuracy. As shown in Figure 7, the red and cyan curves correspond to the outcomes following the application of the shutter correction model in isolation and the combined environmental temperature compensation and shutter correction models, respectively. These corrections substantially mitigated errors induced by temperature variations and shutter effects, enhancing the stability and precision of the data. Despite minor fluctuations in the temperature curve due to ambient temperature changes and shutter correction interventions, the corrected data maintain a consistent average level, with only minor peaks under varying external conditions. The standard deviation of the corrected detector response curve, under continuous temperature fluctuations ranging from 10 °C to 40 °C, was calculated to be 108.175. Utilizing the previously determined radiometric calibration coefficient α = 160.1, the temperature stability was ascertained to be less than 0.676 K (RMES3).

Combined with the comprehensive analysis of the temperature correction error, radiation calibration error, and shutter temperature correction error, and utilizing the previously determined radiometric calibration coefficient α = 160.1, the temperature stability was ascertained to be less than 0.96 K.

(19) $$\frac{\sqrt{RMES1^{2}+RMES2^{2}+RMES3^{2}}}{\alpha }=0.96$$

## 4. Experimental Verification

In practical applications, the performance of infrared gas cloud imaging equipment is primarily determined by two critical metrics: the capability to detect gas leaks in real-world imaging scenarios, and the frequency of false alarms in the absence of leaks. To verify the effectiveness of the temperature model developed for the uncooled gas cloud camera, experiments are conducted to evaluate the improvement effect of the model on these two indicators.

### 4.1. False Detection Rate Test

**Figure 8:**
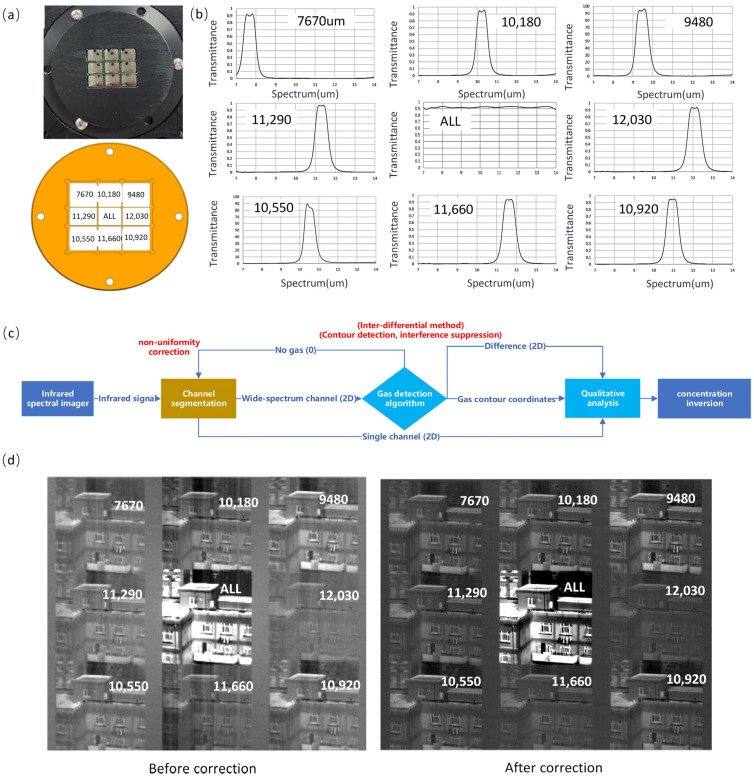
(**a**) Photograph of the dispersive component and spectral filter distribution in the snapshot spectral imaging system, (**b**) measured transmittance curves for each spectral channel, (**c**) flowchart of the gas detection and concentration calculation process, and (**d**) image preprocessing process showing the effects of non-uniformity noise correction. The optical system of the imaging setup is a two-stage imaging system. Mismatched apertures between the primary imaging telescope and the secondary imaging system, along with transmittance inconsistencies between the central and peripheral fields of the lens, lead to signal discrepancies between the center and edges of the image, resulting in envelope noise. Additionally, the infrared detector and imaging circuitry may introduce non-uniformity noise across the image plane. Through correction of a set of external field images, the effectiveness of this process can be visually demonstrated, with the raw image (pre-correction, **left**) showing improved imaging results (post-correction, **right**). In this workflow, the results of gas detection directly impact system performance and are critical to detection sensitivity. The current method used for gas detection is primarily the differential threshold detection method, which detects gas leakage points through temporal differencing and multispectral feature recognition. Improved temperature response stability allows for lower detection thresholds, meaning that the inclusion of the temperature correction model indirectly enhances the detection performance of the equipment.

False detection refers to the system incorrectly detecting a gas leak, that is, the detection equipment mistakenly believes that there is a gas leak when there is actually no leak. A snapshot spectral imaging system was used to conduct verification experiments. The experimental scene is set up as shown in Figure 8.

Snapshot gas spectral imaging system: The snapshot gas spectral imaging system employs a multi-aperture design, integrating several apertures on a single detector plane to achieve simultaneous imaging across multiple spectral bands. Each aperture is equipped with specific bandpass filters or dispersive elements, enabling the system to capture multi-channel spectral data; Figure 8a presents the actual photograph of the multi-aperture optical component (top) and its schematic diagram (bottom). To optimize gas detection, custom filter arrays were designed based on gas absorption spectra, with central wavelengths at 7.67 μm, 1.018 μm, 9.48 μm, 1.129 μm, ALL (refers to the absence of filtering in the long-wave range of 8–14 µm), 1.203 μm, 1.055, 11.66 μm, and 10.92 μm, as can be seen in Figure 8b. Each filter has a full width at half maximum (FWHM) of 0.7 μm, striking a balance between the bandpass-averaged absorbance and the signal-to-noise ratio. The system features an FTII1280 vanadium oxide (VOx) uncooled infrared detector, with a resolution of 1024 × 1280 pixels and a noise equivalent temperature difference (NETD) of 50 mK (at 25 °C, F#1.0). This configuration reduces power consumption, size, and cost while delivering high performance. The optical system achieves a modulation transfer function (MTF) above 0.4 for each field of view, and the RMS radius of the speckle in each field is controlled to be under 10 μm. This precise focus within individual pixels enhances the system’s spectral resolution.

Temperature control: Ambient temperature fluctuations significantly affect the performance of uncooled detectors. To enable quantitative detection, we designed a high-precision temperature control system using thermoelectric cooling (TEC) at the base of the support structure. The target temperature control range was set to ±10 °C with a stability of ±0.1 °C. The entire sensor head was maintained in an airtight condition to ensure the stability of the detector components and the optical system, thereby minimizing the impact of environmental temperature disturbances on the detector’s response. The primary function of temperature control is to regulate the internal temperature variations of the equipment, simulating specific environmental changes based on real weather conditions in Hangzhou (Data are based on historical reanalysis datasets from the European Centre for Medium-Range Weather Forecasts (ECMWF)/National Aeronautics and Space Administration (NASA), provided by www.xihe-energy.com) [19].

Gas Cell: The gas cell, as illustrated in the figure, was constructed from 3.5 mm thick hard aluminum with an airtightness rating of 0.2 MPa and an optical path length of 620 mm. The cell is equipped with germanium glass at both ends, and four circular quartz glass windows on the sides—three for visual inspection and one with a fan to mitigate gas stratification. The top of the cell includes inlets, outlets, and a port for sensor installation.

Electronics: The electronic system comprises four modules: a visible light camera, an uncooled thermal imaging unit, a gimbal system, and a temperature control unit. Each module operates independently, with its drivers and algorithms embedded within the module, providing output data to a higher-level gateway or main control unit. The main control unit handles command relaying and engineering parameter collection, without engaging in the individual functions of each module.

To facilitate the understanding of the relationship between infrared radiation measurements and gas detection, a brief introduction to the equipment’s concentration detection algorithm is necessary. From a data flow perspective, the gas cloud detection algorithm consists of four main components: image segmentation, gas detection, qualitative gas analysis, and concentration inversion. The channel segmentation process includes baseline correction, non-uniformity correction, and channel segmentation methods for infrared images. Once segmentation is complete, the full-channel data are sent to the gas detection module. This module identifies gas contours and removes interference; when gas is detected, it proceeds with gas identification and relevant inversion calculations. The qualitative analysis algorithm determines the type of gas, relying on image data from individual channels. Concentration inversion is used to calculate the concentration within the gas contours, based on the absorption difference caused by the gas and the type of gas. Using Beer’s law, the concentration magnitude is calculated. The flowchart in Figure 8c illustrates the entire data processing workflow.

Prior to concentration calculations, data preprocessing is essential. The spectral camera provides a two-dimensional image from the detector, containing sub-images for each of the nine spectral channels. Each sub-image has an identical size and captures the same scene (excluding spectral variations). The preprocessing involves two main steps: correcting detector non-uniformity noise and performing channel segmentation and image registration.

Firstly, non-uniformity correction parameters are determined through laboratory calibration. By imaging a uniform extended blackbody source, the non-uniform noise magnitude and coordinates for each pixel in the detector are obtained. This calibration process can be represented as follows:

(20) $$Y=X\;\left(x,y\right)+B\;\left(x,y\right)$$

where *Y* is a constant value calculated as the average response of the detector over the blackbody surface, *X*(*x*,*y*) is the actual measured value, and *B*(*x*,*y*) denotes the offset for each detector pixel. The effectiveness of the correction is illustrated in Figure 8d.

Channel segmentation and registration aim to achieve the pixel-level alignment of spatial–spectral three-dimensional data. This process involves two steps: localizing interior orientation elements and matching image feature points. Initially, the primary point of the interior orientation and image distortion parameters are used for a preliminary extraction of image content. Subsequently, feature points within the image are matched to achieve sub-pixel registration accuracy.

In the presence of gas in the detection field of view, absorption occurs, resulting in a change in irradiance, denoted as ∆L, which can be expressed as follows:

(21) $$∆L=L_{I}-L_{gas}=L_{I}\;\left(1-\tau_{gas}\right)$$

where $L_{I}$ is the radiance in the absence of gas, $L_{gas}$ is the radiance with gas present, and $\tau_{gas}$ is the gas transmittance. The relationship between $L_{I}$ and the DN values is determined by radiometric calibration, and within a specific wavelength range, they are nearly linearly related (depending on the detector’s response linearity). Therefore, the relationship can also be represented as follows:

(22) $$∆DN=DN-DN_{gas}=DN\left(1-\tau_{gas}\right)$$

where DN, $DN_{gas}$, and ∆DN are the detector DN values in the absence of gas, no gas, and the resulting difference, respectively. Once the device measures the DN values, we can determine ∆L and thus obtain $T_{gas}$.

According to the Beer–Lambert–Bouger law, this change in irradiance is related to gas concentration and the optical path of the gas within the field of view. For moderate gas concentrations, where the absorption cross-section is stable, τ_gas can be approximated as follows:

(23) $$\tau_{gas}=e^{-\alpha_{gas}CL}$$

where $\alpha_{gas}$ is the gas absorption coefficient, C is the gas concentration, and L is the optical path length of the gas. This approximation allows us to derive the gas concentration C based on the detected irradiance changes.

In summary, calculating the gas concentration involves measuring the change in transmittance along the observation path caused by the presence of gas, which is then used to determine the concentration. The DN values obtained by the equipment enable the estimation of the radiance change, transmittance calculation, and subsequent application of the Beer–Lambert–Bouguer law to derive the gas concentration. Since the optical path length L of the gas cloud is typically challenging to obtain in real-time during measurements, concentration is expressed in units of ppm-m, representing the gas concentration per unit optical path length.

**Figure 9:**
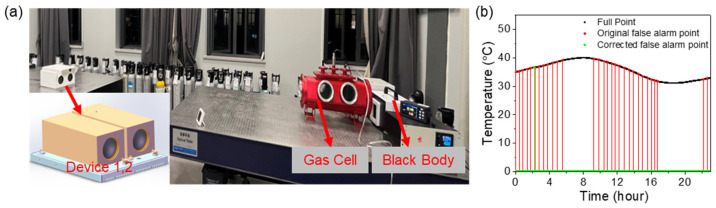
(**a**) Experimental scene construction. (**b**) False alarm test results.

To realistically simulate field conditions, we used temperature records from Hangzhou on 1 August to emulate the diurnal temperature variations. In Figure 9a, we set up an experimental scenario in the laboratory to simulate temperature variations. The setup includes two infrared imaging devices, one for conducting the gas monitoring experiment and the other for the control experiment. A gas cell was used to store gases of different concentrations and types. To quantitatively control the background radiation level of the gas, a blackbody was placed behind the gas cell. The gas cell was vacuum-sealed, containing no gases. Two multi-aperture imaging systems were employed simultaneously, both using the same temperature scheme and data acquisition protocol. Both systems were equipped with online image processing and gas leak alarm capabilities. The primary difference was that Device A incorporated the temperature correction model developed in this study, whereas Device B did not. To facilitate the long-term statistical analysis of alarm data, the alarm function was configured to terminate automatically after 5 min. The experiment ran for 24 h.

The results in Figure 9b show that the gas cloud detection equipment with the temperature compensation model has an overall stable performance in the temperature changes of the day, with only one false alarm occurring in the morning when the temperature changes rapidly. In contrast, in a group of experiments without the compensation model, a total of 30 false alarms occurred in one day.

### 4.2. Detection Limit of Gas Concentration

To assess the influence of the temperature prediction model on the lower detection limits of gas concentrations in gas cloud imaging, a series of experiments were conducted using a gas cell containing various gases, including sulfur hexafluoride, ethylene, cyclohexane, and ammonia. The experimental design comprised two groups: one utilized the compensation model for calibration, while the control group operated without any calibration. By comparing the inversion errors of gas concentrations between the two groups, we evaluated the effectiveness of the correction model in enhancing detection limits. In the experimental setup, a constant-temperature blackbody was positioned on one side of the gas cell to ensure thermal stability, while the imaging system was placed on the opposite side at a specified distance to collect infrared response data. Initially, the gas cell was purged with nitrogen and subsequently evacuated to near vacuum using a pump. Following this, the imaging system recorded the response signal in the absence of gas. Different concentrations of the target gases were then introduced sequentially, and response data were collected for each channel at standard concentrations of 0.5×, 1×, and 1.5×, with variations in atmospheric pressure introduced to increase data diversity.

The results indicated that the gas cloud detection devices equipped with the correction model effectively reduced the detection error and improved the lower limit detection capability. For instance, in the SF6 tests, the device incorporating the temperature compensation model detected a concentration of 500 ppm (equivalent to 750 ppm-m) at 1.5 atm, whereas the reference device only registered the gas at a concentration of 1500 ppm-m. In the case of methane, which is typically challenging to detect, the device with the temperature compensation model accurately measured a concentration of 3375 ppm, while the control group exhibited no response throughout the testing period. Overall, the detection limits of SF6, ethylene, cyclohexane, and ammonia were improved by 50%, 33%, 25%, and 67%, respectively (see Table A1 in Appendix A).

## 5. Conclusions

### 5.1. Summary

This paper presented a temperature compensation model for industrial applications, which enables accurate infrared radiation prediction across a broad operating temperature range without the need for stringent equipment temperature control, achieving a detection accuracy within 0.96 °C. Moreover, due to the stability of the equipment under ambient temperature, the integration of this temperature compensation model into the gas cloud detection system resulted in improvements in the lower detection limits for SF6, ethylene, cyclohexane, and ammonia by 50%, 33%, 25%, and 67%, respectively.

### 5.2. Error Analysis

When analyzing the error sources of this method, the first factor to consider is the error in the temperature measurement calibration process. In experiments, a blackbody is typically used as the temperature standard and stable light source, which is a common and feasible approach. However, when there are large temperature fluctuations in the experimental environment (such as in a thermal chamber with a temperature range of 10 °C to 40 °C), the error of the blackbody may increase. This is because the continuous temperature changes in the thermal chamber can affect the stability of the blackbody, introducing noise and causing uncertainty in the calibration process.

In addition to blackbody error, the noise inherent in the infrared detector is also a significant factor affecting the accuracy of temperature calibration. Although the temperature drift characteristics of the detection system have been successfully modeled during calibration, the detector is still influenced by circuit noise, photon noise, and the thermal noise inherent to uncooled detector materials. These noises typically exhibit characteristics of white noise. Even with noise suppression techniques such as multi-frame accumulation and digital TDI, some residual noise persists, which further impacts the accuracy of temperature measurement.

## Figures and Tables

**Table 1:** Equipment and performance parameters.

Manufacturers	IRay
Detector type	Uncooled surface detector
Array size	1280 × 1024
Sensitivity NETD	<50 mK (F/1300 K, 50 Hz)
F-number	1.0
Pixel spacing	12 µm
Power consumption	~2.5 W

## Data Availability

Data are contained within the article.

## Appendix A

**Table A1:** Comparison of Gas Concentration Detection Limits for Two Devices.

Gas Type	Standard Concentration (ppm-m)	Atmospheric Pressure in Gas Cell (atm)	Equivalent Concentration (ppm-m)	Device A (Adjust the Lower Threshold Limit to 5 DN)	Concentration Calculation for Device A (ppm-m)	Device A Predicted Concentration Lower Limit (ppm-m *)	Device B: (Default Lower Threshold 20 DN)	Concentration Calculation for Device B (ppm-m)	Device B Predicted Concentration Lower Limit (ppm-m)	Lower Limit Improvement Effect (%)
Sulfur hexafluoride	500	0.5	250	0	-	750	0	-	1500	50%
	500	1	500	0	-		0	-		
	500	1.5	750	1	890		0	-		
	1000	0.5	500	0	-		0	-		
	1000	1	1000	1	1231		0	-		
	1000	1.5	1500	1	1432		1	573		
	1500	0.5	750	1	874		0	-		
	1500	1	1500	1	1529		1	633		
	1500	1.5	2250	1	2317		1	1757		
Ethylene	1000	0.5	500	0	-	2000	0	-	3000	33%
	1000	1	1000	0	-		0	-		
	1000	1.5	1500	0	-		0	-		
	2000	0.5	1000	0	-		0	-		
	2000	1	2000	1	2083		0	-		
	2000	1.5	3000	1	2690		1	2986		
	3000	0.5	1500	0	-		0	-		
	3000	1	3000	1	2890		1	2555		
	3000	1.5	4500	1	4535		1	3984		
Methane	750	0.5	375	0	-	3375	0	-	-	-
	750	1	750	0	-		0	-		
	750	1.5	1125	0	-		0	-		
	1500	0.5	750	0	-		0	-		
	1500	1	1500	0	-		0	-		
	1500	1.5	2250	0	-		0	-		
	2250	0.5	1125	0	-		0	-		
	2250	1	2250	0	-		0	-		
	2250	1.5	3375	1	1931		0	-		
cyclohexane	1200	0.5	600	0	-	1800	0	-	2400	25%
	1200	1	1200	0	-		0	-		
	1200	1.5	1800	1	452		0	-		
	2400	0.5	1200	0	-		0	-		
	2400	1	2400	1	1980		1	2005		
	2400	1.5	3600	1	3424		1	3111		
	3600	0.5	1800	1	689		0	-		
	3600	1	3600	1	3545		1	3277		
	3600	1.5	5400	1	5897		1	5286		
Ammonia	900	0.5	450	1	213	450	0	-	1350	67%
	900	1	900	1	655		0	-		
	900	1.5	1350	1	1209		1	989		
	1800	0.5	900	1	546		0	-		
	1800	1	1800	1	1434		1	1560		
	1800	1.5	2700	1	2654		1	2546		
	2700	0.5	1350	1	1090		1	1278		
	2700	1	2700	1	2567		1	2467		
	2700	1.5	4050	1	4269		1	4253		

* ppm-m: ppm-m represents “parts per million per meter”. It is used to describe the cumulative effect of gas concentration over a given path length, reflecting the total amount of gas over a one-meter path.
